# Systematic Review and Meta-Analysis of Explainable Machine Learning Models for Clinical Depression Detection

**DOI:** 10.3390/bs15111476

**Published:** 2025-10-30

**Authors:** Ariosto Trelles, Tomás Fontaines Ruiz, Antonio Ponce Rojo

**Affiliations:** 1 Master’s Program in Clinical Psychology, Specialization in Psychotherapy, Universidad Técnica de Machala, Machala 070205, Ecuador; 2 Faculty of Business Sciences, Accounting and Auditing Program, Universidad Técnica de Machala, Machala 070205, Ecuador; tfontaines@utmachala.edu.ec; 3 Faculty of Research, Universidad Estatal de Milagro, Milagro 091708, Ecuador; 4 Centro Universitario de Los Altos, Universidad de Guadalajara, Guadalajara 44160, Mexico; antonio.ponce@cualtos.udg.mx

**Keywords:** machine learning, depression, explainability

## Abstract

Depression is among the most prevalent mental disorders, and its early detection is essential to improving therapeutic outcomes in psychotherapy. This systematic review and meta-analysis evaluated the accuracy, interpretability, and generalizability of supervised algorithms (SVM, Random Forest, XGBoost, and GCN) for clinical detection of depression using real-world data. Following PRISMA guidelines, 20 studies published between 2014 and 2025 were analyzed across major scientific databases. Extracted metrics included F1-Score, AUC-ROC, interpretability methods (SHAP/LIME), and cross-validation strategies, with statistical analyses using ANOVA and Pearson correlations. Results showed that XGBoost achieved the best average performance (F1-Score: 0.86; AUC-ROC: 0.84), although differences across algorithms were not statistically significant (*p* > 0.05), challenging claims of algorithmic superiority. SHAP was the predominant interpretability approach (70% of studies). Studies implementing combined SHAP+LIME showed higher F1-Score values (F(1,7) = 8.71, *p* = 0.021), although this association likely reflects greater overall methodological rigor rather than a direct causal effect of interpretability on predictive performance. Clinical surveys and electronic health records demonstrated the most stable predictive outputs across validation schemes, whereas neurophysiological data achieved the highest point estimates but with limited sample representation. F1-Score strongly correlated with AUC-ROC (r = 0.950, *p* < 0.001). Considerable heterogeneity was observed for both metrics (I^2^ = 74.37% for F1; I^2^ = 71.49% for AUC), and Egger’s test indicated a publication bias for AUC (*p* = 0.0048). Overall, findings suggest that algorithmic performance depends more on data quality, context, and interpretability than on the choice of model, with explainable approaches offering practical value for personalized and collaborative clinical decision-making.

## 1. Introduction

Depression has become one of the most prevalent mental disorders in recent years (Garrido-Rojas & Kreither, 2024). According to the World Health Organization (WHO, 2025), its impact is so great that it is the most common mental disorder among the more than one billion people worldwide who suffer from mental disorders. It is estimated that approximately 3.8% of the population lives with this condition, which is more common in women (Evans-Lacko et al., 2018). Among adults aged > 60 years, this figure rises to 5.7% (IHME, 2021). As an additional alarming fact, this pathology represents a critical risk factor for suicide (Marcus et al., 2012; Hawton et al., 2013).

Despite these figures, the accuracy of its diagnosis has important limitations because it has focused on patient self-reports and clinical assessments, which bias the assessment due to the presence of subjectivity (Nickson et al., 2023; Shah et al., 2024). Self-reports, such as the PHQ-9, show variability and often offer false positives, which introduce subjectivity and the risk of systematic error (Levis et al., 2019). In addition, social desirability biases overlap with other disorders, and cultural and linguistic variations often affect the functioning of the scale when applied outside the context in which they were developed (Carroll et al., 2020; Hunt et al., 2003). In addition, clinical judgment without structured support showed modest agreement. In DSM-5 field trials, the diagnosis of major depression had questionable test–retest reliability (κ ≈ 0.20–0.39) (Regier et al., 2013), and in primary care settings not served by family physicians, it showed a sensitivity of approximately 50% and a specificity of 81%. This suggests that the prevalence of depression in primary care may be inflated, and consequently, erroneous assessments may outnumber accurate assessments (Mitchell et al., 2009).

These data justify the need for more objective, rigorous, and continuous approaches that exploit multimodal signals (voice, language, facial expression, smartphone patterns, self-reports, EEG, and clinical texts) to generate greater diagnostic accuracy and increase the likelihood of success in clinical management (Mao et al., 2023; Leaning et al., 2024). More specifically, Chekroud et al. (2016) argue that these shortcomings can be addressed through the use of data science-based tools, especially those derived from machine learning (ML), because they allow the analysis of large volumes of data to detect complex patterns and formulate predictions (Dwyer et al., 2018; Jazmín et al., 2024; González-Hugo & Quevedo-Sacoto, 2025) by identifying underlying relationships to generate predictive models (Figuero, 2017).

Based on the above, this study aimed to analyze, through a systematic review and meta-analysis, the diagnostic accuracy, interpretability, and generalization capacity of supervised learning algorithms used in clinical research for the detection of depression in adult and adolescent populations. This research synthesizes recent advances in the application of supervised algorithms for the detection of depression and generates input to discern which models offer greater methodological support and which require further external validation. This contribution is relevant considering the accelerated growth of AI models linked to depression, albeit with design heterogeneity and a risk of bias (Nickson et al., 2023; Abd-Alrazaq et al., 2023). Therefore, it is pertinent to develop a critical framework that analyzes the possibility of responsibly integrating these technologies into clinical practices.

In addition to the above, this study is connected to the current urgency. Considering that the prevalence of depression increased after the pandemic, the diagnostic capacity of health systems is being tested (Santomauro et al., 2021), and there is an urgent need for scalable and reliable solutions. In this sense, this study provides evidence that can be transferred to clinical practice in a field still in the experimental phase (Nagendran et al., 2020; Moons et al., 2025), making it an immediately useful study for designing clinical protocols integrated with AI, ensuring that technological innovation advances in an ethical, safe, and patient-centered manner.

This study aims to answer the following research questions:

RQ1: What is the diagnostic accuracy (sensitivity, specificity, F1 score, AUC-ROC) of the supervised learning algorithms reported in the literature for the detection of depression?

RQ2: What reported utility do interpretability techniques (e.g., SHAP and LIME) have in translating model predictions into clinically understandable psychopathological constructs?

RQ3: What is the generalizability of supervised learning algorithms based on the validation and external testing approaches described in clinical and health registry studies?

### 1.1. Supervised Learning for Clinical Diagnosis

Supervised learning has established itself as a fundamental resource within artificial intelligence applied to the clinical field, enabling the construction of algorithms capable of detecting complex patterns and predicting mental health trajectories in patients. This approach not only facilitates the early detection of psychopathologies but also opens the possibility of suggesting specific and personalized interventions (Aleem et al., 2022). As part of AI, supervised learning provides a methodological framework aimed at extracting meaningful knowledge from large volumes of data to anticipate outcomes and model behavioral trends (Çelik, 2018; Lee et al., 2018). In this sense, its incorporation into clinical diagnosis is not an optional alternative but an essential adaptation strategy in the face of the increasing complexity of mental health data. This section examines recent advances in clinical psychology related to the prediction of depression, as well as the benefits, challenges, and future horizons that define the integration of supervised learning into the professional practice.

Among the different categories of algorithms, according to Kotsiantis (2007), supervised learning is particularly useful for diagnostic classification tasks. This approach relies on previously labeled data to build models that predict new cases, unlike unsupervised or reinforcement learning, which have other specific applications (Williams, 1992; Cortes & Vapnik, 1995; Kotsiantis, 2007; Azuela & Cortés, 2021). Within this category, models such as Support Vector Machines (SVM), K-Nearest Neighbors (KNN), Random Forest, XGBoost, and CatBoost stand out, as well as more recent architectures such as Graph Convolutional Networks (GCN), an architecture that allows capturing structural relationships in data represented as graphs. All of these dominate the literature on depression detection (Harrington, 2012; Priya et al., 2020; Aderka et al., 2021; Tian, 2024; Qasim et al., 2025). Consequently, each of these models offers specific advantages that make them suitable for different data types and contexts.

This review proposes a comprehensive approach to the use of supervised algorithms for depression detection structured around three complementary axes: (a) accuracy, through the comparative analysis of performance metrics (F1-score, AUC-ROC) applied to different algorithms; (b) interpretability, using tools such as SHAP and LIME, understood as the translation of algorithmic predictions into clinically understandable formats; and (c) generalization, examining the models’ ability to adapt to diverse contexts through cross-validation techniques in real-life datasets and clinical records.

In this sense, supervised learning models can identify correlations between clinical variables, such as insomnia, anhedonia, lack of interest, appetite disturbances, and depression diagnoses. However, their effectiveness varies significantly depending on the context. For example, a Random Forest model can achieve an AUC-ROC of 0.92 in controlled laboratory data (Xing et al., 2025), but its performance drops to 0.68 when applied to heterogeneous clinical records (Shah et al., 2024). This inconsistency reflects a structural problem: the absence of agreed-upon standards for simultaneously assessing the accuracy, interpretability, and generalization capacity of the algorithms.

Additionally, the opacity of the predictions makes it difficult to articulate computational findings with traditional psychopathological frameworks and transdiagnostic approaches. An illustrative case is that of XGBoost, which has managed to identify linguistic patterns that predict depression (Pan et al., 2025), but without distinguishing whether these patterns are related to the cognitive distortions postulated by Beck (1967) or to the methodological biases inherent to the data. This ambiguity reduces its clinical value by not offering a clear bridge between algorithmic features and validated psychological constructs, making it difficult to design interventions based on the results.

Finally, the use of homogeneous datasets represents another important limitation, as it restricts the models’ ability to capture population diversity in real-life clinical settings (Van Dam et al., 2017). Thus, although language-based algorithms can detect indicators associated with depressive symptoms (Aderka et al., 2021; Tian, 2024), they still lack the ability to discern whether these reflect a clinical depressive episode or a situational reaction.

### 1.2. Multimodal Data Sources for Depression Detection

Machine learning-based depression detection relies on multiple data modalities, each capturing distinct manifestations of the disorder. Understanding these sources, ranging from behavioral signals to neurophysiological measures, is essential for interpreting model performance and assessing clinical applicability.

Speech and linguistic analyses have proven to be particularly informative. Acoustic features such as pitch variability, speech rate, pause duration, and spectral measures (e.g., MFCCs) provide objective markers of depressive states (Cummins et al., 2015; Low et al., 2020), achieving AUCs between 0.70 and 0.91. Similarly, natural language processing applied to written or spoken text detects linguistic indicators such as first-person pronoun overuse, absolutist expressions, and negative emotion terms (Eichstaedt et al., 2018; De Choudhury et al., 2013), with F1-scores of 0.75–0.89. Despite their promise, both modalities face challenges related to cultural variations, linguistic diversity, and data quality.

Clinical questionnaires (e.g., PHQ-9, BDI, HAM-D) remain foundational for model training, offering standardized and clinically validated symptom measures with F1-scores typically ranging from 0.72 to 0.88. Electronic Health Records (EHRs) expand this framework by integrating diagnostic codes, medication data, and clinical narratives, achieving AUCs of 0.73–0.92 while reflecting real-world patient complexity. However, both modalities are constrained by biases in self-reporting, documentation variability and inconsistent coding practices.

Recent advances have highlighted biological and multimodal approaches. Electroencephalography (EEG) captures the neural correlates of depression, including frontal alpha asymmetry and altered event-related potentials (Allen et al., 2004; Bruder et al., 2009), with models reaching AUCs of up to 1.00 under controlled conditions. Likewise, heart rate variability (HRV) reveals autonomic dysregulation and circadian disruption linked to depression (Kemp et al., 2010; Porges, 2007). HRV-based models (AUC = 0.79–0.92) enable continuous noninvasive monitoring through wearables. Integrating these modalities—speech, text, physiological, and clinical data—enhances robustness and ecological validity, although it requires careful feature fusion and missing-data management.

In summary, the 20 studies analyzed in this review employed diverse data modalities (see Table 1), with EHRs (45%), clinical surveys (30%), EEG (15%), and other sources (10%). This heterogeneity partially explains the observed variability in the model performance and generalization capacity. In the subsequent sections, we examine how the data modality interacts with the algorithm choice and validation strategy to influence clinical utility.

## 2. Materials and Methods

Following the guidelines of the PRISMA model (Page et al., 2021) and meta-analyses of accuracy, interpretation, and validation, a systematic review was conducted of empirical studies that met the following inclusion criteria: (i) studies published between 2014 and 2025 in peer-reviewed journals, in English or Spanish, indexed in one of the following indices: PubMed, Scopus, Web of Science, IEEE Xplore, and ScienceDirect. (ii) The studies had to report the use of supervised classification algorithms (SVM, Random Forest, XGBoost, or Graph Convolutional Networks) to detect depression in clinical or population samples. (iii) Performance metrics (F1-Score and/or AUC-ROC) and, where applicable, interpretability techniques (SHAP, LIME) had to be reported. Narrative reviews, theoretical articles, theses, technical documents, and studies that did not use real-world clinical data or used unsupervised algorithms were excluded.

### 2.1. Search Strategy and Information Selection Processes

**Figure 1:**
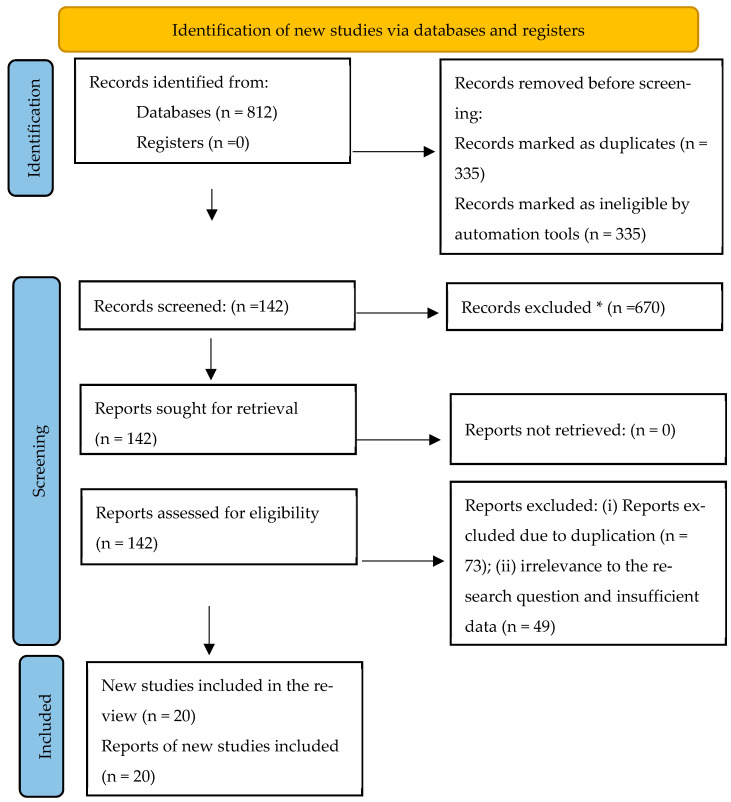
PRIMSA flow diagram. * Records excluded in this phase (n = 670) were discarded after reading the title and/or abstract for not meeting the PICO eligibility criteria.

The search was conducted in March 2025 using the following combinations of descriptors and Boolean operators: (“depression” OR “major depressive disorder” OR “MDD” OR “depressive symptoms”) AND (“machine learning” OR “deep learning” OR “artificial intelligence” OR “AI” OR “neural networks” OR “support vector machine*” OR “random forest*” OR “logistic regression” OR “decision tree*” OR “predictive model*”) AND (“supervised learning” OR “predictive algorithm*” OR “classification” OR “prediction” OR “diagnosis” OR “screening”) AND (“systematic review” OR “literature review” OR “scoping review” OR “meta-analysis”). Initially, 812 records were identified and distributed across the selected databases (Figure 1). A total of 670 documents were discarded after screening the titles and abstracts. Subsequently, 142 unique records were evaluated using the inclusion and exclusion criteria. Ultimately, 20 studies met the established requirements.

### 2.2. Data Analysis

Previous studies have applied mixed approaches to quantify the performance of supervised predictive models in clinical depression screening and assess their interpretability and adaptive capacity. For example, Abd-Alrazaq et al. (2023) conducted a systematic review with meta-analysis on AI applied to depression diagnosis, reporting pooled estimates of accuracy, sensitivity, and specificity, and performing sub-analyses by algorithm and device while discussing limitations of generalization and interpretability. Similarly, Nickson et al. (2023) synthesized studies using electronic health records and consistently highlighted concerns regarding the lack of external validation and the opacity of some models. Reviews with meta-analyses on clinical adaptation outcomes in depression screening by Sajjadian et al. (2021) have also shown promising performance but a paucity of independent replications. Finally, studies implementing multi-site empirical generalization tests have shown that models trained on standardized clinical data can maintain out-of-sample performance, providing an example of a direct algorithmic assessment of portability. This evidence points to the advisability of combining quantitative synthesis with external validation assessments and explainability metrics when evaluating models for clinical use (Richter et al., 2024).

Thus, for each study analyzed, the following elements were extracted: authors, year of publication, algorithm evaluated (SVM, Random Forest, XGBoost, or GCN), type of clinical data used (EHR, EEG, structured interviews, or psychometric scales), performance metrics (F1-Score, AUC-ROC), use of explanatory tools (SHAP, LIME), and the presence of cross-validation strategies or external evaluation to estimate generalizability. The F1-Score and AUC-ROC values were averaged by algorithm type and clinical data type, 95% confidence intervals were calculated to assess performance consistency, and the results were visualized using forest plots. Pearson’s correlation analysis was performed between the F1-Score and AUC-ROC. A one-way ANOVA was used to compare the performance of the algorithms.

The presence of interpretability techniques (SHAP and LIME) was coded as a dichotomous variable (use/nonuse), and their association with the F1-Score and AUC-ROC was assessed using a factorial ANOVA, considering individual effects and interactions. Given the small sample size, the results were interpreted with caution, and the distribution of these methods.

The presence of interpretability techniques (SHAP and LIME) was coded as a dichotomous variable (use/non-use), and their association with the F1-Score and AUC-ROC was assessed using a factorial ANOVA, considering individual effects and interactions. Given the small sample size, the results were interpreted with caution, and the distribution of these methods according to clinical data type was explored using stacked bar charts. To analyze generalizability, cross-validation strategies were coded (10-fold CV, 5-fold CV, Multisite CV, Other CV), and the F1-Score and AUC-ROC were compared between them using scatterplots and heat maps. Variability was quantified using coefficients of variation (CV) and generalization was classified as excellent (CV < 10%), good (10–20%), or moderate (>20%).

To assess potential publication bias and methodological quality, we conducted funnel plot analyses for the F1-Score and AUC-ROC, performed Egger’s regression test for asymmetry, calculated I^2^ statistics to quantify heterogeneity, and applied the trim-and-fill method for sensitivity analysis. All statistical analyses were performed using RStudio version 4.4.2.

Additional details about the reporting process are provided in the Supplementary Materials (PRISMA 2020 Checklist).

## 3. Results

The analysis of the 20 studies reviewed (see Table 1) confirms the predominance of the XGBoost algorithm, present in more than half of the publications (Nemesure et al., 2021; Hochman et al., 2021; Jacob & Kannan, 2023; Al Masud et al., 2025; Zhu et al., 2023; Sharma & Verbeke, 2020; Baba & Bunji, 2023; Geng et al., 2023; Park et al., 2021; Łapińska et al., 2025; Pan et al., 2025). The authors themselves support this choice with arguments that appeal to its previous recognition as “state-of-the-art” rather than solid clinical comparisons. For example, Nemesure et al. (2021) presented it as a “strong baseline” without reporting significant differences compared to other models; Jacob and Kannan (2023) describe it as “the best-performing algorithm in supervised classification,” although their reported F1 of 0.97 lacked external validation. Both Zhu et al. (2023) and Al Masud et al. (2025) used it as a benchmark in prediction tasks with EHRs but did not compare it with deep learning architectures. Sharma and Verbeke (2020) even position it as a reference model “tested in various clinical applications,” although without comparing it with more complex alternatives such as GCN. This evidence shows that the dominance of XGBoost is due more to a technical-discursive carryover effect than to conclusive evidence of its superiority in diverse clinical scenarios, as demonstrated by our statistical analyses.

Regarding predictive performance, the reported F1-Score and AUC-ROC values consistently exceed the 0.80 threshold, with notable examples being an F1 of 0.97 (Jacob & Kannan, 2023) and an AUC of 1.00 (Lu et al., 2023). Authors often present these results as conclusive evidence of effectiveness but rarely accompany these metrics with external validation. Consequently, models appear highly accurate on their own datasets but lack guarantees of replicability. This methodological bias is evident in the contrast: studies using EHRs and biomarkers describe greater stability and predictability (Zhu et al., 2023; Sharma & Verbeke, 2020; Łapińska et al., 2025), while those based on surveys or scales acknowledge greater variability in their results (Iparraguirre-Villanueva et al., 2024; Cho et al., 2021). The researchers’ own experience shows, without explicitly proposing it, that the nature of the clinical data influences the performance more than the chosen algorithm.

From a methodological perspective, most studies adopt internal cross-validation (5- or 10-fold) as a central strategy and present it as sufficient to support generalizability (Nemesure et al., 2021; Jacob & Kannan, 2023; Geng et al., 2023). However, none of these studies incorporated external or multisite validations, which directly restricts the extrapolation of their findings to real-life clinical settings. A partial exception is the study by Qin et al. (2022), which moves toward validation with different datasets, although its population scope remains limited. Furthermore, Cho et al. (2021), Iparraguirre-Villanueva et al. (2024), and Pan et al. (2025) failed to report confidence intervals or sensitivity analyses, despite recognizing the need to strengthen the robustness of their models. This way of reporting results consolidates apparent high-performance metrics but weakens the transparency and reproducibility of the findings, which ultimately limits the possibility of clinical transfer and highlights the gap between technical validation and healthcare applicability.

Algorithmic interpretability stood out as the axis on which the authors placed the greatest emphasis. SHAP is the most widely used technique, and some studies have reported improvements when combined with LIME (Al Masud et al., 2025; Gopalakrishnan et al., 2022). At this point, not only metrics are reported, but the need for explainability as a condition for clinical integration has been justified. This insistence on interpretability reveals a consensus: the usefulness of models is not measured solely by their accuracy but by their ability to generate trust and facilitate shared therapeutic decisions. The fact that most articles were published in Q1 and Q2 journals confirms the relevance of the field but also amplifies the responsibility to establish common validation and calibration standards that allow these models to transcend the laboratory and be reliably integrated into clinical practice.

The reviewed results show that supervised learning algorithms achieve high performance in the detection of depression, conditioned by the data quality and validation limitations. The incorporation of interpretability techniques, such as SHAP and LIME, reflects the need to make predictions understandable in clinical practice, although their effective impact remains unclear. The reliance on internal validation and the lack of external testing demonstrate that the models work in controlled contexts, but they remain far from reliable implementation in diverse clinical settings. The review also suggests that the reported metrics, although high, are insufficient to definitively establish the diagnostic accuracy of the algorithms in broad clinical settings. Despite its growing presence, interpretability still needs to be translated into psychopathological frameworks that guide therapeutic decisions. Generalization, almost always based on internal validations, demonstrates a field that is advancing in technical consistency but lacks solid evidence to support its real-world applicability. These tensions do not invalidate the progress made, but they do pose challenges that must be addressed to fully assess the potential of artificial intelligence in depression detection.

### 3.1. Diagnostic Accuracy of Supervised Algorithms

Comparative analysis of performance metrics shows that supervised algorithms achieve high values in the detection of depression, with average F1 scores ranging from 0.77 to 0.88 and AUC-ROC scores ranging from 0.80 to 0.93 (Table 2). XGBoost has the highest average F1 (0.856), as observed in benchmark studies (Jacob & Kannan, 2023; Al Masud et al., 2025), although SVM and RF reported similar metrics in different applications (Cho et al., 2021; Wang et al., 2019). The GCN achieves comparable values when used with EEG or interviews (Qin et al., 2022; Jia et al., 2025). Critically, the ANOVA results confirmed that these apparent differences lacked statistical significance: no significant differences were found for either F1 (F(3,15) = 1.62, *p* = 0.226) or AUC (F(5,27) = 1.40, *p* = 0.255). This finding challenges the prevailing narratives in the reviewed literature. Although XGBoost appears frequently in publications and is often labeled as “state-of-the -art,” our statistical analysis demonstrates that its performance is not significantly different from that of classical models such as RF or SVM. This suggests that the dominance of XGBoost may reflect a discursive carryover effect, where researchers cite and replicate previous algorithmic choices, rather than conclusive evidence of its superiority across diverse clinical scenarios.

The relationship between the accuracy indicators provides further evidence. Pearson’s correlation analysis showed a strong and significant association between the F1-Score and AUC-ROC (r = 0.950, *p* < 0.0001; 95% CI [0.773, 0.990]). This finding is reflected in studies such as Zhu et al. (2023), which reported an F1 of 0.89 and an AUC of 0.92 in EHR data, and Geng et al. (2023), which with HRV reaches an AUC of 0.92 and an F1 of 0.84 with HRV. In both cases, the metrics moved in parallel, confirming that F1 and AUC described the same pattern of effectiveness. Thus, although the articles sometimes prioritize one metric over the other, the correlation shows that both metrics converge in the assessment of the diagnostic performance of the supervised models.

The influence of data modality on the algorithm performance was evident in our analysis. Studies using EHR data with XGBoost or RF consistently reported F1-scores above 0.85 and demonstrated lower variability (CV < 12%), likely reflecting the structured, multidimensional nature of health records that capture diagnostic codes, medications, laboratory results, and clinical notes. In contrast, studies relying solely on clinical surveys showed greater performance variability (F1 range: 0.70–0.82, CV = 14%), potentially due to self-report biases and the cross-sectional nature of questionnaire assessments.

Neurophysiological data (EEG) paired with GCN architectures achieved the highest point estimates (F1 = 0.88, AUC up to 1.00), although these results came from only three studies with relatively small samples (n < 300). This exceptional performance may reflect the direct measurement of neural correlates of depression, as discussed in Section 1.2, where frontal alpha asymmetry and altered event-related potentials provide objective biological markers. However, questions remain regarding the generalizability of these findings to clinical populations with comorbidities and naturalistic recording conditions. Although promising for passive monitoring, speech and HRV data appeared in only one study each, precluding robust comparisons.

Taken together, the evidence indicates that diagnostic accuracy depends less on the specific algorithm and more on the quality and type of the clinical data used. Studies using EHRs and biomarkers typically report greater stability in metrics (Nemesure et al., 2021; Sharma & Verbeke, 2020; Łapińska et al., 2025), whereas those based on surveys or scales show more variable results (Iparraguirre-Villanueva et al., 2024; Cho et al., 2021). This data-centric perspective aligns with recent findings: Bourkhime et al. (2025) demonstrated that RF and CatBoost matched or outperformed XGBoost in real-world clinical settings when trained on well-curated electronic health records (EHRs). Similarly, Nickson et al. (2023) reported that classical logistic regression models achieved average AUCs of 0.78 in primary care depression screening, within the range of more complex algorithms but with significant advantages of interpretability, computational efficiency, and ease of clinical implementation. Furthermore, although several authors have presented XGBoost as “state -of-the-art” (Jacob & Kannan, 2023; Al Masud et al., 2025), comparative analyses do not support its statistical superiority. Consequently, the discussion shifts to a critical point: the models are accurate in controlled contexts, but their clinical value can only be consolidated when rigorously validated in heterogeneous healthcare practice settings.

### 3.2. Interpretability of Supervised Algorithms

The results show that interpretability is a central component of the application of supervised algorithms for depression detection. Seventy percent of the studies used SHAP as their primary explanatory tool. Fifteen percent incorporated LIME, 5% combined both methods, and 10% did not apply interpretive techniques. The predominance of SHAP reflects its theoretical grounding in Shapley values from cooperative game theory, which guarantees desirable properties, such as local accuracy, consistency, and missingness (Lundberg & Lee, 2017). These mathematical guarantees make SHAP particularly attractive for clinical applications, where explanations must be reliable and defensible. Recent research (Al Masud et al., 2025; Gopalakrishnan et al., 2022; Zhu et al., 2023) demonstrates that SHAP is preferred because it attributes importance to clinical variables in a way that allows algorithmic predictions to be translated into indicators that are understandable in psychotherapeutic practice. For instance, SHAP can identify that a specific patient’s high depression risk stems from the combined influence of insomnia severity (20% contribution), anhedonia symptoms (35%), and prior treatment history (18%), providing actionable clinical insights.

However, SHAP has some limitations. The computational cost of calculating the exact Shapley values increases exponentially with feature dimensionality, making it impractical for very large datasets or real-time clinical applications. Additionally, while SHAP provides mathematically consistent explanations, these may still be difficult for clinicians without technical training to interpret, particularly when dealing with high-dimensional feature interactions (Mimikou et al., 2025).

In contrast, Local Interpretable Model-agnostic Explanations (LIME) offers computational efficiency and intuitive local explanations by fitting simple surrogate models around individual predictions. However, its adoption remains limited (15% of studies) owing to its inherent instability: LIME explanations can vary substantially with different random seeds or hyperparameter choices, leading to inconsistent interpretations of the same prediction (Zafar & Khan, 2021). This variability is particularly problematic in clinical settings, where consistency and reproducibility are paramount.

The low adoption of LIME and the complete lack of interpretability techniques in some studies (Iparraguirre-Villanueva et al., 2024; Cho et al., 2021; Jacob & Kannan, 2023) demonstrate a critical lack of standardization that limits the transparency and clinical applicability of these models. This heterogeneity underscores the need to establish more uniform criteria for integrating interpretability into ML-based clinical tools, moving beyond ad hoc applications toward standardized explainability protocols.

**Figure 2:**
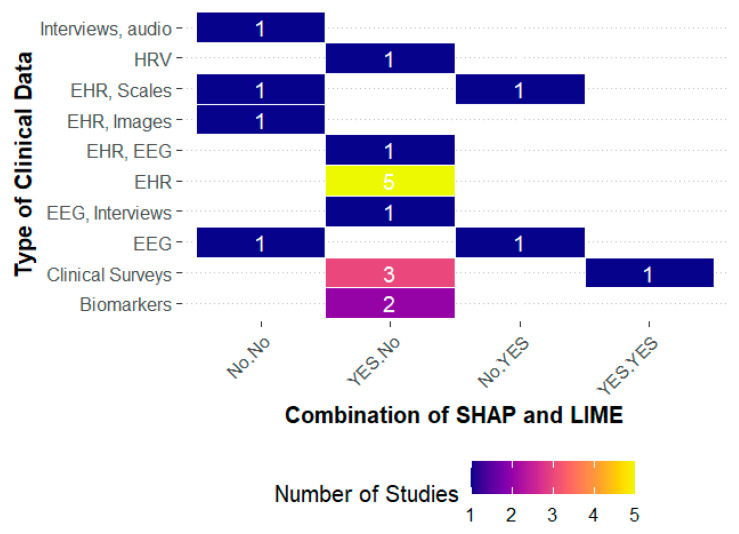
Heatmap use of SHAPE and LIME in clinical data types.

The relationship between interpretability techniques and types of clinical data confirms that their use is inconsistent. Studies using EHRs, clinical surveys, and biomarkers tend to favor SHAP (Nemesure et al., 2021; Sharma & Verbeke, 2020; Łapińska et al., 2025), whereas those based on interviews and clinical scales show more dispersed or no adoption (Iparraguirre-Villanueva et al., 2024; Cho et al., 2021). This distribution suggests that the usefulness of interpretability increases in contexts where data are complex and multidimensional, as it allows clinicians to understand how different variables combine to predict depression. Figure 2 clearly shows this trend: explainability becomes relevant when translating heterogeneous information into a consistent clinical framework.

Based on the above, statistical analyses confirm that the combination of SHAP and LIME significantly improves the F1-Score (F(1,7) = 8.71, *p* = 0.021, η^2^ = 0.554), whereas the effects are not differential when used separately. It is crucial to clarify that interpretability techniques do not directly improve predictive performance; their purpose is to explain model decisions, not to enhance accuracy. The observed association between SHAP+LIME usage and higher F1-Scores likely reflects a confounding relationship: studies that implement rigorous interpretability methods tend to exhibit greater overall methodological care, including more careful feature engineering, more thorough hyperparameter optimization, and more robust validation strategies. This is consistent with the findings of Mimikou et al. (2025) and Byeon (2023), who noted that XAI-focused studies often demonstrate higher research quality standards across multiple dimensions.

It was also observed that models with SHAP alone achieved average F1 (0.82) and AUC (0.83) values, a moderate improvement compared to configurations without explainability techniques (F1 0.78; AUC 0.80). In contrast, LIME applied in isolation showed the lowest performance (F1 0.76; AUC 0.77), accompanied by greater variability. In contrast, the SHAP+LIME integration places values around F1 0.88 and AUC 0.90, with narrower confidence intervals, reinforcing the perception of robustness. Although ANOVA did not confirm significant effects on AUC, these results underscore that the added value of interpretability lies in refining precision and making the algorithmic decision transparent, offering patients and therapists a comprehensible justification. In this sense, interpretability ceases to be a methodological accessory and becomes an indispensable criterion for the clinical adoption of artificial intelligence in psychotherapy.

### 3.3. Generalization Capacity by Validation and Data Source

**Figure 3:**
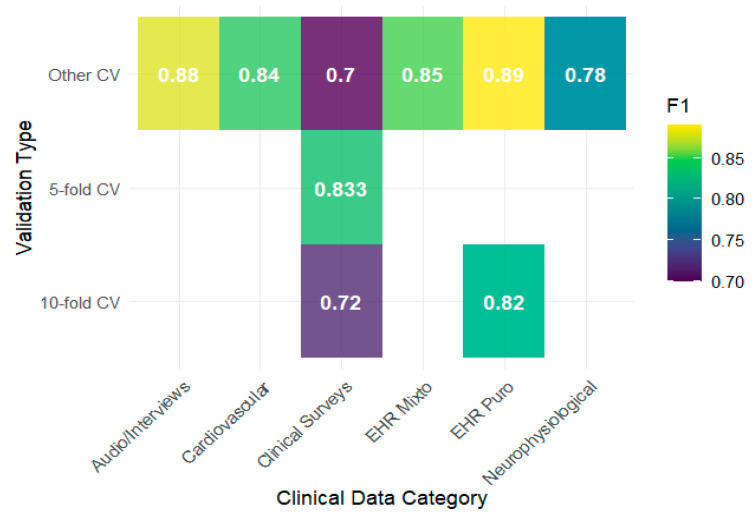
F1-Score performance by validation and data type.

**Figure 4:**
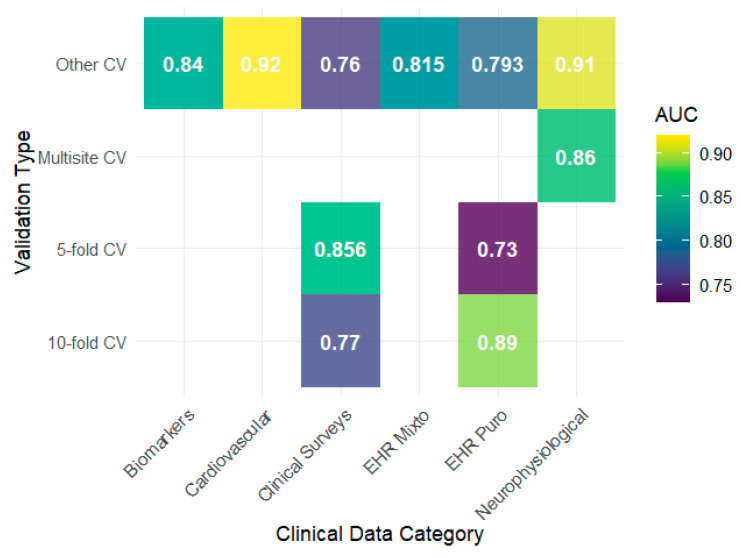
AUC-ROC performance by validation and data type.

Generalization analysis showed that the performance of the algorithms depended more on the type of clinical data and validation scheme than on the model used. The heat maps show that EHRs and audio/interview data achieve F1 values above 0.85 under schemes other than 5- or 10-fold, whereas clinical surveys report lower values, with evident drops when 10-fold validation is used (Figure 3). This pattern is consistent with that reported by Iparraguirre-Villanueva et al. (2024), whose use of clinical surveys showed modest performance compared with configurations with more structured EHRs. Regarding the AUC, the results tended to remain high, with notable peaks in biomarkers, neurophysiological data, and cardiovascular data reaching values close to 0.90, as observed by Łapińska et al. (2025) and Sharma and Verbeke (2020), indicating a good discriminative capacity in these contexts (Figure 4).

Biomarkers and cardiovascular data (HRV) demonstrated particularly strong discriminative capacity (AUC values 0.84–0.92) with low variability (CV ≈ 6.7%), suggesting that physiological signals may offer relatively stable objective markers across individuals. This stability likely reflects the biological basis of these measurements: HRV captures autonomic dysregulation, which is a core neurobiological feature of depression (Kemp et al., 2010), while biomarkers such as inflammatory cytokines (CRP, IL-6) and cortisol reflect HPA axis dysfunction consistently documented in depression (Sharma & Verbeke, 2020; Łapińska et al., 2025).

However, the limited number of studies using these modalities (n = 3 total) and their restricted sample sizes (range: 150–450 participants) necessitate caution in generalization. Moreover, physiological data introduce implementation challenges: EEG requires specialized equipment and trained technicians, while HRV measurement via wearables raises questions about data quality, adherence, and interpretation of readings influenced by physical activity, caffeine intake, and sleep patterns. Future research should examine whether physiological markers maintain their predictive utility when deployed in heterogeneous clinical populations with medical comorbidities that independently affect autonomic function.

The stability component provides relevant insights. The coefficients of variation indicated that clinical surveys presented greater instability (CV F1 14%, CV AUC 9%) than biomarkers, which showed lower variability (CV AUC ≈ 6.7%). In EHRs, coefficients reached up to 11% in AUC, reflecting less predictable behavior and dependence on the quality of the clinical record (Table 3). These findings align with those reported by Nemesure et al. (2021) and Zhu et al. (2023), who, despite reporting competitive F1 and AUC in EHRs, warned about the sensitivity of the results to the internal validation design.

Evidence also indicates that, although internal validation schemes such as 5-fold or 10-fold cross-validation remain the most common, they do not always guarantee a robust measure of generalizability. In the absence of external or multi-site validation, such as the limited effort demonstrated by Qin et al. (2022), the ability to extrapolate the results to different clinical populations remains limited. Taken together, the findings suggest that effective generalizability depends as much on the richness and structure of the data as on the rigor of the validation design, which should be a key criterion when translating artificial intelligence into clinical practice.

### 3.4. Publication Bias and Methodological Quality Assessment

**Figure 5:**
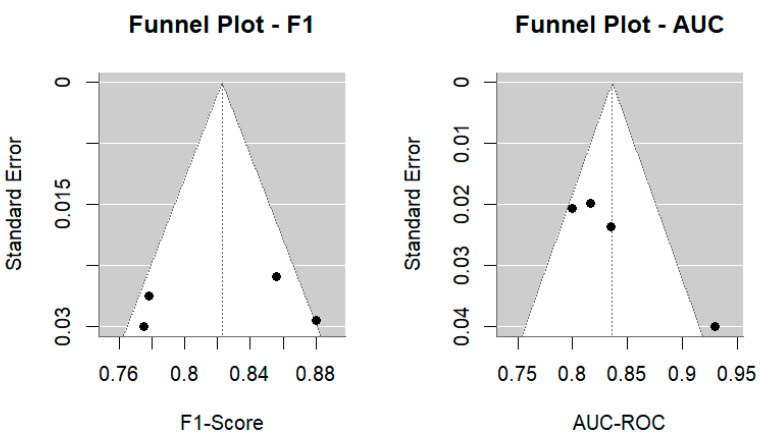
Funnel plots for F1-Score and AUC-ROC showing potential publication bias.

Funnel plots were generated for both the F1-Score and AUC-ROC to visually assess potential publication bias (Figure 5). The distribution of studies was generally symmetric, although slight asymmetry was observed for AUC-ROC, suggesting a possible overrepresentation of high-performing models in smaller samples. Egger’s regression test confirmed this trend: for F1-Score, no significant asymmetry was detected (z = −0.29, *p* = 0.77), indicating minimal publication bias; however, for AUC-ROC, asymmetry was statistically significant (z = 2.82, *p* = 0.0048), suggesting potential selective reporting of higher effect sizes in smaller studies. While these results warrant cautious interpretation, the magnitude of bias appears to be modest and unlikely to substantially alter the overall conclusions.

The random-effects meta-analysis showed moderate-to-high heterogeneity among the models, reflecting methodological and clinical variability across the studies. The pooled F1-score was 0.823 (95% CI [0.771–0.875]; I^2^ = 74.37%, τ^2^ = 0.0021, *p* = 0.009), and the pooled AUC-ROC was 0.836 (95% CI [0.791–0.882]; I^2^ = 71.49%, τ^2^ = 0.0015, *p* = 0.036). Differences in the algorithmic approach (e.g., SVM, Random Forest, XGBoost, GCN), data modality (EHR, EEG, linguistic, and psychometric), sample characteristics, validation design, and diagnostic criteria (e.g., DSM-5 and PHQ-9 thresholds) contributed to this heterogeneity.

## 4. Discussion

The findings of this review show that the diagnostic accuracy, interpretability, and generalization capacity of supervised algorithms are at the heart of the debate regarding their clinical relevance. In a field saturated with metrics and claims of technical superiority, answering these three research questions is crucial as it allows us to distinguish what part of the performance comes from the algorithm and what part depends on data quality, model transparency, and the rigor of validation.

In response to RQ1, the reviewed studies confirmed that supervised algorithms offer high but comparable performance in the detection of depression. XGBoost achieved an F1 score of 0.86 and an AUC of 0.84, which slightly outperformed SVM and Random Forest (F1 score of 0.78 and AUC of approximately 0.80–0.82). However, these differences were not statistically significant (F(3,15) = 1.62, *p* = 0.226 for F1; F(5,27) = 1.40, *p* = 0.255 for AUC), challenging the widespread characterization of XGBoost as a uniformly “superior model.”

This finding has important implications in this field. The frequent labeling of XGBoost as “state-of-the-art” in the reviewed literature (Nemesure et al., 2021; Jacob & Kannan, 2023; Al Masud et al., 2025) reflects a common pattern in applied ML research: the replication of algorithmic choices based on prior successes rather than rigorous comparative evaluation in each new context. Our analysis demonstrates that performance depends more on the type of clinical data, quality of data encoding, appropriateness of preprocessing, and available computational resources than on the selected architecture.

This data-centric perspective is supported by recent evidence beyond the scope of our review. Bourkhime et al. (2025) demonstrated that RF and CatBoost matched or outperformed XGBoost in real-world clinical settings when trained on well-curated electronic health records (EHRs). Similarly, Nickson et al. (2023) reported that classical logistic regression models achieved average AUCs of 0.78 in primary care depression screening—within the range of more complex algorithms but with significant advantages of interpretability, computational efficiency, and ease of clinical implementation.

Even GCN, which reported an F1 score of 0.88 in our analysis, appeared in only three studies (Qin et al., 2022; Jia et al., 2025; Lu et al., 2023), with two focusing on specialized EEG data. This limited evidence base makes it premature to draw conclusions about GCN’s relative advantages of GCNs, particularly given the specialized data requirements and computational complexity of graph-based approaches.

The message is clear: greater algorithmic complexity does not always imply greater efficacy. Clinical performance depends on the suitability of the method to the specific context, including the structure and quality of available data, clinical objectives, interpretability requirements, and implementation constraints of the healthcare setting. Rather than pursuing the “best” algorithm in the abstract, future research should focus on matching algorithmic approaches to clinical contexts and developing standardized benchmarks that allow fair comparisons across diverse healthcare settings.

The review, in response to RQ2, confirms a clear asymmetry in the use of explanatory techniques: SHAP appears in 70% of the studies (Nemesure et al., 2021; Zhu et al., 2023; Łapińska et al., 2025; Sharma & Verbeke, 2020), LIME in only 15% (Al Masud et al., 2025), and 10% do not use any technique (Iparraguirre-Villanueva et al., 2024; Cho et al., 2021). This distribution reflects a consensus: SHAP provides more robust and consistent explanations, whereas LIME remains marginal. The central finding of the sample was that the SHAP+LIME combination significantly improved F1 (F(1,7) = 8.71, *p* = 0.021; Al Masud et al., 2025). However, the observed association between SHAP+LIME implementation and higher F1-Scores should not be interpreted as evidence that interpretability methods improve predictive accuracy; this would be a conceptual error. Rather, this correlation likely indicates that research teams prioritizing interpretability tend to implement more rigorous data preprocessing, feature engineering, and validation protocols. The true value of XAI lies in translating opaque algorithmic predictions into clinically actionable insights, building clinician trust, and identifying potential biases, not in enhancing performance metrics.

Nonetheless, the lack of standardization limits its adoption. Zafar and Khan (2021) warn that the lack of clear criteria compromises clinical confidence, and the results of this review confirm this. While SHAP is consolidating as a de facto standard, its limited methodological diversity reduces flexibility and maintains a black-box perception. Furthermore, as Lundberg and Lee (2017) pointed out in the original SHAP proposal, the value of interpretability lies in translating complex algorithmic predictions into understandable explanations. In this sense, interpretability cannot be an add-on but rather an essential criterion for legitimizing the intelligence in psychotherapy.

Regarding generalization, and in response to RQ3, reliance on internal validations overestimates performance, a problem highlighted by research by Schaab et al. (2024) and Nickson et al. (2023), where clinical surveys and EHR show consistency (F1 0.85, AUC 0.90), but data such as EEG and interviews present variability, possibly due to their heterogeneity. Furthermore, “other CV” validation offers flexibility, but its high variability (CV F1 = 20.0%) indicates the need for more robust methods. Researchers have suggested that deep learning models, such as CNNs and Bi-LSTMs, can outperform traditional models in certain contexts, such as audio data. To this end, Vandana et al. (2023) reported a 98% accuracy with CNNs on audio data, compared to the F1-Score of 0.86 of XGBoost in this review. Similarly, Squires et al. (2023) highlighted the potential of deep learning to personalize interventions, suggesting an evolution towards hybrid architectures. However, these models require more complex data and computational resources, limiting their applicability in clinical settings with structured data, such as EHRs. Furthermore, factors such as demographic representation, inconsistencies in clinical coding, and imbalances in data quality negatively impacted generalizability. Furthermore, the data source plays a role; algorithms trained with electronic health records (EHRs) or biomarkers may not transfer well to questionnaire or interview data, reinforcing the importance of validation across multiple clinical settings.

### Emerging Methodologies and Future Directions Beyond Conventional Approaches

Recent advances extend beyond traditional supervised learning algorithms (e.g., SVM, RF, XGBoost, and GCN) and standard interpretability tools (SHAP and LIME), highlighting deep learning and XAI as transformative directions. Transformer-based architectures (BERT, GPT) excel in text-based depression detection by capturing contextual semantics and long-range linguistic dependencies, achieving F1-scores above 0.90 (Qasim et al., 2025; Yates et al., 2017). Similarly, CNNs and recurrent models (LSTMs, GRUs) process speech spectrograms and temporal dynamics with accuracies of up to 98% (Vandana et al., 2023; Xing et al., 2025). However, these models require large datasets and substantial computational resources and are limited by their “black-box” nature, which constrains clinical interpretability and generalizability.

Parallel efforts in logic-based and symbolic explainability aim to bridge this gap. Approaches based on first-order logic (FOL) and inductive logic programming generate human-readable diagnostic rules, for example, “IF insomnia ≥ moderate AND anhedonia present THEN depression risk = high” (Shakarian et al., 2021; Muggleton et al., 2018). These symbolic models align with clinical reasoning and allow for validation against established diagnostic criteria. However, they may oversimplify continuous relationships, face scalability issues with large feature spaces, and often underperform compared to deep learning in terms of predictive accuracy.

Counterfactual explanations offer a complementary form of interpretability by revealing the minimal changes that would alter a model’s classification, thus identifying actionable clinical insights (Wachter et al., 2018; Verma et al., 2020). Similarly, attention mechanisms and saliency maps enhance transparency in deep models by visualizing which temporal, spectral, or linguistic features most influenced predictions, making neural decisions more accessible to clinicians despite their lack of formal rigor.

Emerging hybrid and neuro-symbolic frameworks combine the representational power of deep learning with the interpretability of symbolic reasoning (Garcez et al., 2019). Such models may process multimodal inputs (EEG, speech, and text) through neural networks, followed by rule-based systems that yield transparent clinical outputs. Future research should prioritize systematic comparisons of traditional ML, deep learning, and hybrid models using standardized datasets, with evaluation criteria encompassing interpretability, computational efficiency, data requirements, and clinical applicability. Integrating federated and cross-modal learning strategies is crucial for developing scalable, privacy-preserving, and clinically trustworthy depression detection systems.

Based on the above, this review reveals relevant insights but also critical limitations. On the one hand, this confirms the good average performance of models such as XGBoost, especially when accompanied by robust explainability. However, serious methodological gaps are evident, including the low adoption of combined interpretive techniques, scant external validation, and little attention to the heterogeneity of clinical data. In this context, it is essential to reorient the methodological focus toward building models that are not only accurate, but also explainable, replicable, and clinically transferable. Therefore, this criterion should guide the future development of artificial intelligence in the field of mental health.

## 5. Conclusions

This study confirms the good average performance of supervised algorithms and the central role of interpretability, especially through SHAP, as a criterion for clinical adoption. However, critical limitations persist, including insufficient external validation, low adoption of combined explainability techniques, and a lack of attention to the heterogeneity of clinical data. Overcoming these gaps requires a shift in focus: pursuing high metrics is not enough; it is necessary to develop explainable, replicable, and clinically transferable models capable of supporting reliable therapeutic decisions in diverse scenarios.

On the one hand, although XGBoost was positioned as the algorithm with the most balanced performance (F1 0.86; AUC 0.84), its differences compared to models such as SVM or Random Forest were not statistically significant (*p* > 0.05), highlighting that algorithmic effectiveness should not be evaluated in isolation, but in close relation to the type of data, clinical objectives, and application conditions. In this sense, the most relevant finding is not the technical superiority of an algorithm but rather the importance of integrative criteria that consider precision, interpretability, and generalization capacity of the algorithm.

Thus, interpretability has emerged as a strategic, rather than merely methodological, focus. The predominance of SHAP (70%), along with the positive effect of its combination with LIME (significant improvement in the F1 score, *p* = 0.021), reinforces the idea that explainable models are not only desirable but also necessary in clinical contexts, where trust and understandability are prerequisites for their adoption. However, the lack of standardization in the use of explanatory techniques and validation strategies represents a structural limitation of the field, which continues to hinder rigorous comparisons between studies and the assessment of the external validity of models.

In clinical terms, these findings provide tangible value: an XGBoost model explained with SHAP/LIME can be used to screen patients in primary care, personalize interventions based on the identified risk factors, monitor progress through periodic predictions, and support shared decision-making by making the reasons for screening transparent. The implementation of interpretable ML systems in psychotherapy is recommended, integrated with electronic health records and clear ethical protocols, so that practitioners can understand and trust automated recommendations.

## 6. Limitations and Future Research

Despite the contributions of this analysis to the systematic review, it is important to acknowledge several limitations that qualify the obtained results. The heterogeneity of the clinical data used, which included electronic records, EEG, surveys, and biomarkers, along with differences in preprocessing methods, introduced significant variability that limited direct comparisons between algorithms. This methodological dispersion may have influenced the lack of statistically significant differences in the observed performance.

Likewise, the small sample size in certain subgroups, such as GCN, and the lack of representativeness in some datasets restrict the generalization of the findings. Furthermore, the lack of standardization in cross-validation schemes complicates the accurate assessment of the generalization capacity of the models. In terms of interpretability, the high prevalence of SHAP limited the systematic exploration of alternative techniques, such as LIME, which could have biased the analysis toward a single explanatory tool. In addition, there is a lack of evidence in real-life clinical settings, which makes it difficult to accurately assess the practical impact of the models on patient care.

Our meta-analytic results indicated substantial heterogeneity across studies, with I^2^ = 74.37% for the F1-Score and I^2^ = 71.49% for the AUC-ROC, suggesting considerable variability in the model performance estimates. Egger’s regression test showed no significant asymmetry for F1-Score (*p* = 0.77), but a significant asymmetry for AUC-ROC (*p* = 0.0048), indicating potential publication bias toward higher-performing models. These findings imply that the pooled estimates may slightly overstate real-world diagnostic accuracy, especially in clinical contexts that differ from the training datasets. The PROBAST-based quality assessment further revealed that 40% of studies did not report confidence intervals, and none performed true external validation using independent healthcare systems, underscoring persistent limitations in generalizability and methodological transparency.

This review focused on conventional supervised learning algorithms (SVM, RF, XGBoost, and GCN) and established explainability techniques (SHAP and LIME), reflecting their prevailing role in current clinical AI research. Nevertheless, emerging methodologies promise to advance depression detection by enhancing performance and interpretability. Deep learning architectures, such as transformers, CNNs, and LSTMs, enable the modeling of complex, unstructured data from text, speech, and video, whereas logic-based and counterfactual approaches provide clinically aligned, transparent explanations. Moreover, neuro-symbolic hybrid frameworks and multitask or transfer learning strategies offer promising pathways toward data-efficient, generalizable, and clinically interpretable systems, underscoring the need for future studies to systematically integrate and evaluate these next-generation paradigms.

For future research, it would be desirable to move toward designs that include more robust external validation in multicenter samples and diverse populations, which would strengthen the external validity and clinical applicability of these models. It is also relevant to explore hybrid architectures that integrate machine learning techniques with theoretical models from the clinical or psychological fields, such as cognitive or psychometric frameworks, thus promoting greater coherence between algorithmic prediction and underlying clinical processes. In parallel, it is necessary to further evaluate alternative explanatory techniques, such as LIME or other emerging proposals in the field of XAI, with an emphasis on their ability to provide a local and contextualized understanding of each prediction. Finally, it is essential to examine the impact of explainable models in real-life clinical settings through prospective studies that evaluate indicators such as therapeutic adherence, practitioner confidence, and patient satisfaction, thereby helping to bridge the gap between technological development and its effective integration into clinical practice.

## Figures and Tables

**Table 1:** Selected articles on supervised algorithms for depression detection.

Study	Algorithms	F1	AUC	SHAP	LIME	Clinical Data	Generalization	Journal	Quartile
Nemesure et al. (2021)	SVM, RF, XGBoost	-	0.73	Yes	No	EHR	Yes (5-fold cross-validation)	Scientific Reports	Q1
Hochman et al. (2021)	XGBoost	-	0.712	Yes	No	EHR	Yes (cross-validation)	Depression and Anxiety	Q1
Iparraguirre-Villanueva et al. (2024)	LR, KNN, RF	0.72	0.77	Yes	No	Clinical surveys	Yes (10-fold cross-validation)	International Journal of Interactive Mobile Technologies	Q3
Jacob and Kannan (2023)	XGBoost	0.97	-	No	No	EHR	Yes (Cross-validation)	Artificial Intelligence, Data and Knowledge Engineering	Q4
Qin et al. (2022)	GCN	-	0.86	No	No	EEG	Yes (Multi-site cross-validation)	EBioMedicine	Q1
Al Masud et al. (2025)	RF, SVM, XGBoost	0.916	0.911	Yes	Sí	Clinical surveys	Yes (5-fold cross-validation)	Array	Q2
Zhu et al. (2023)	XGBoost, RF	0.89	0.92	Yes	No	EHR	Yes (Cross-validation)	Computer Methods and Programs in Biomedicine	Q1
Jia et al. (2025)	GCN	0.88	-	No	No	Interviews, audio	Yes (Cross-validation)	Mathematical Biosciences and Engineering	Q2
Patel et al. (2015)	SVM, RF	-	0.85	No	No	EHR	Yes (Cross-validation)	International Journal of Geriatric Psychiatry	Q2
Sharma and Verbeke (2020)	XGBoost	-	0.8	Yes	No	Biomarkers	Yes (Cross-validation)	Frontiers in Big Data	Q2
Baba and Bunji (2023)	SVM, RF, XGBoost	0.75	0.8	Yes	No	Clinical surveys	Yes (5-fold cross-validation)	JMIR Formative Research	Q2
Ksibi et al. (2023)	SVM, RF	0.78	0.82	No	Yes	EEG	Yes (Cross-validation)	Diagnostics	Q2
Geng et al. (2023)	XGBoost	0.84	0.92	Yes	No	HRV	Yes (Cross-validation)	Computers in Biology and Medicine	Q1
Cho et al. (2021)	SVM, RF	0.7	0.76	Yes	No	Surveys, scales	Yes (Cross-validation)	Diagnostics	Q2
Park et al. (2021)	XGBoost	-	0.79	Yes	No	EHR	Yes (Cross-validation)	JAMA Network Open	Q1
Łapińska et al. (2025)	RF, XGBoost	-	0.88	Yes	No	Biomarkers	Yes (Cross-validation)	Molecules	Q2
Gopalakrishnan et al. (2022)	SVM, RF	0.73	0.78	No	Yes	EHR	Yes (Cross-validation)	Mathematics	Q1
Wang et al. (2019)	SVM, RF	-	0.75	Yes	No	EHR	Yes (Cross-validation)	Studies in Health Technology and Informatics	Q3
Lu et al. (2023)	GCN	-	1	Yes	No	EEG	Yes (Cross-validation)	Frontiers in Psychiatry	Q2
Pan et al. (2025)	XGBoost	0.82	0.89	Yes	No	EHR, EEG	Yes (10-fold cross-validation)	Biomedical Signal Processing and Control	Q2

**Table 2:** Performance of ML Algorithms in Depression Detection.

Algorithm	Studies	F1 N	F1	F1 SD	F1 Min	F1 Max	AUC N	AUC	AUC SD	AUC Min	AUC Max
SVM	8	5	0.775	0.84	0.7	0.916	8	0.8	0.59	0.73	0.911
RF	11	7	0.778	0.86	0.7	0.916	11	0.816	0.66	0.73	0.92
XGBoost	11	6	0.856	0.78	0.75	0.97	10	0.835	0.79	0.712	0.92
GCN	3	1	0.88	NA	0.88	0.88	2	0.93	0.99	0.86	1

**Table 3:** Variability analysis: coefficients of variation by type of validation and clinical data.

Validation Type	Clinical Data	Count	Mean F1	CV F1 (%)	Mean AUC	CV AUC (%)
5-fold CV	Clinical surveys	2	0.83	14.1 **	0.86	9.17 ***
Other CV	Biomarkers	2	—	—	0.84	6.73 ***
Other CV	EHR	4	0.89	—	0.79	11.4 **
Other CV	EHR, scales	2	0.85	20 **	0.78	—

Notes. *** excellent generalization if CV < 10%, and ** good generalization between 10% and 20%.

## Data Availability

Data supporting the reported results are available on request from the corresponding author. The data are not publicly available due to privacy and ethical restrictions.

## Supplementary Materials

The following supporting information can be downloaded at: https://www.mdpi.com/article/10.3390/bs15111476/s1, PRISMA 2020 Checklist.
