# Supplementary material for: Systematic Review and Meta-Analysis of Explainable Machine Learning Models for Clinical Depression Detection
Source: Behav Sci (Basel). 2025 Oct 30;15(11):1476. doi: 10.3390/bs15111476 (PMC12649417; doi:10.3390/bs15111476)
Supplement: Supplementary file 1 [file behavsci-15-01476-s001.zip › behavsci-3904922-supplementary.pdf]

PRISMA 2020 Checklist

Section and Topic	Item #	Checklist item	Location where item is reported
**TITLE**			
Title	1	Identify the report as a systematic review.	Title page - "Systematic Review and Meta-Analysis of Explainable Machine Learning Models for Clinical Depression Detection"
**ABSTRACT**			
Abstract	2	See the PRISMA 2020 for Abstracts checklist.	Abstract section - Includes background, methods (PRISMA guidelines, databases, inclusion/exclusion criteria), results (20 studies, performance metrics), and conclusions
**INTRODUCTION**			
Rationale	3	Describe the rationale for the review in the context of existing knowledge.	Introduction, paragraphs 2-4 - Discusses limitations of current depression diagnosis methods, need for objective approaches, and role of ML
Objectives	4	Provide an explicit statement of the objective(s) or question(s) the review addresses.	End of Introduction - Three research questions (RQ1: diagnostic accuracy, RQ2: interpretability utility, RQ3: generalizability)
**METHODS**			
Eligibility criteria	5	Specify the inclusion and exclusion criteria for the review and how studies were grouped for the syntheses.	Section 2, paragraph 1 - Studies 2014-2025, supervised algorithms (SVM, RF, XGBoost, GCN), and performance metrics required. Exclusions: narrative reviews, theoretical articles, theses
Information sources	6	Specify all databases, registers, websites, organisations, reference lists and other sources searched or consulted to identify studies. Specify the date when each source was last searched or consulted.	Section 2, databases: PubMed, Scopus, Web of Science, IEEE Xplore, and ScienceDirect
Search strategy	7	Present the full search strategies for all databases, registers and websites, including any filters and limits used.	Section 2.1 - Boolean search strategy provided: ("depression" OR "major depressive disorder"...) AND ("machine learning"...) AND ("supervised learning"...
Selection process	8	Specify the methods used to decide whether a study met the inclusion criteria of the review, including how many reviewers screened each record and each report retrieved, whether they worked independently, and if applicable, details of automation tools used in the process.	Partially reported in Section 2.1 - Mentions 812 initial records, 670 discarded after title/abstract screening, 142 evaluated, and 20 included. Missing: number of reviewers, independence
Data collection process	9	Specify the methods used to collect data from reports, including how many reviewers collected data from each report, whether they worked independently, any processes for obtaining or confirming data from study investigators, and if applicable, details of automation tools used in the process.	Section 2.2, paragraph 2 - Lists extracted elements (authors, algorithms, data types, metrics, interpretability tools). Missing: number of reviewers, independence
Data items	10a	List and define all outcomes for which data were sought. Specify whether all results that were compatible with each outcome domain in each study were sought (e.g. for all measures, time points, analyses), and if not, the methods used to decide which results to collect.	Section 2.2 - F1-Score and AUC-ROC as primary performance metrics. Interpretability (SHAP/LIME) and generalizability (cross-validation) as secondary outcomes
	10b	List and define all other variables for which data were sought (e.g. participant and intervention characteristics, funding sources). Describe any assumptions made about any missing or unclear information.	Section 2.2 - Algorithm type, clinical data type, publication year, journal quartile extracted. Missing: assumptions about missing data
Study risk of bias assessment	11	Specify the methods used to assess risk of bias in the included studies, including details of the tool(s) used, how many reviewers assessed each study and whether they worked independently, and if	Not explicitly reported - Missing: risk of bias assessment methodology

Section and Topic	Item #	Checklist item	Location where item is reported
		applicable, details of automation tools used in the process.	
Effect measures	12	Specify for each outcome the effect measure(s) (e.g. risk ratio, mean difference) used in the synthesis or presentation of results.	Section 2.2 - Mean F1-Score and AUC-ROC with 95% confidence intervals, correlation analysis, ANOVA for algorithm comparison
Synthesis methods	13a	Describe the processes used to decide which studies were eligible for each synthesis (e.g. tabulating the study intervention characteristics and comparing against the planned groups for each synthesis (item #5)).	Partially in Section 2.2 - Studies grouped by algorithm type and clinical data type for analysis
	13b	Describe any methods required to prepare the data for presentation or synthesis, such as handling of missing summary statistics, or data conversions.	Section 2.2 - F1-Score and AUC-ROC averaged by algorithm type, confidence intervals calculated
	13c	Describe any methods used to tabulate or visually display results of individual studies and syntheses.	Section 2.2 - Forest plots, scatterplots, heat maps
	13d	Describe any methods used to synthesize results and provide a rationale for the choice(s). If meta-analysis was performed, describe the model(s), method(s) to identify the presence and extent of statistical heterogeneity, and software package(s) used.	Section 2.2 - ANOVA, Pearson correlations, factorial ANOVA. RStudio 4.4.2 used for analysis
	13e	Describe any methods used to explore possible causes of heterogeneity among study results (e.g. subgroup analysis, meta-regression).	Section 2.2 - Analysis by clinical data type and algorithm type to explore heterogeneity
	13f	Describe any sensitivity analyses conducted to assess robustness of the synthesized results.	Section 2.2 - Coefficients of variation calculated for generalization analysis
Reporting bias assessment	14	Describe any methods used to assess risk of bias due to missing results in a synthesis (arising from reporting biases).	Not reported - Missing: publication bias assessment
Certainty assessment	15	Describe any methods used to assess certainty (or confidence) in the body of evidence for an outcome.	Not reported - Missing: certainty assessment (e.g., GRADE)
**RESULTS**			
Study selection	16a	Describe the results of the search and selection process, from the number of records identified in the search to the number of studies included in the review, ideally using a flow diagram.	Section 2.1 and Figure 1 - 812 records were identified, 670 were discarded, 142 were evaluated, and 20 were included. PRISMA flow diagram provided
	16b	Cite studies that might appear to meet the inclusion criteria, but which were excluded, and explain why they were excluded.	Not explicitly reported - Missing: examples of excluded studies with reasons
Study characteristics	17	Cite each included study and present its characteristics.	Table 1 - Complete characteristics table of all 20 included studies
Risk of bias in studies	18	Present assessments of risk of bias for each included study.	Not reported - Missing: risk of bias assessments
Results of individual studies	19	For all outcomes, present, for each study: (a) summary statistics for each group (where appropriate) and (b) an effect estimate and its precision (e.g. confidence/credible interval), ideally using structured tables or plots.	Table 1 & Table 2 - Individual study results and summary statistics by algorithm presented
Results of syntheses	20a	For each synthesis, briefly summarise the characteristics and risk of bias among contributing studies.	Section 3 & 3.1 - Study characteristics summarized. Missing: risk of bias summary

Section and Topic	Item #	Checklist item	Location where item is reported
	20b	Present results of all statistical syntheses conducted. If meta-analysis was done, present for each the summary estimate and its precision (e.g. confidence/credible interval) and measures of statistical heterogeneity. If comparing groups, describe the direction of the effect.	Table 2 & Section 3.1 - Mean performance metrics with standard deviations, ANOVA results ($F(3,15)=1.62$, $p=0.226$ for F1; $F(5,27)=1.40$, $p=0.255$ for AUC)
	20c	Present results of all investigations of possible causes of heterogeneity among study results.	Sections 3.1-3.3 - Analysis by algorithm type, clinical data type, and validation methods
	20d	Present results of all sensitivity analyses conducted to assess the robustness of the synthesized results.	Table 3 - Coefficients of variation analysis for generalization assessment
Reporting biases	21	Present assessments of risk of bias due to missing results (arising from reporting biases) for each synthesis assessed.	Not reported - Missing: publication bias assessment
Certainty of evidence	22	Present assessments of certainty (or confidence) in the body of evidence for each outcome assessed.	Not reported - Missing: certainty assessment
**DISCUSSION**			
Discussion	23a	Provide a general interpretation of the results in the context of other evidence.	Section 4 - Comprehensive discussion comparing results with existing literature
	23b	Discuss any limitations of the evidence included in the review.	Section 6 - Detailed limitations section covering heterogeneity, sample sizes, validation methods
	23c	Discuss any limitations of the review processes used.	Section 6 - Discusses limitations in methodology including lack of standardization, external validation
	23d	Discuss implications of the results for practice, policy, and future research.	Section 5 (Conclusions) & Section 6 - Clinical implications and future research recommendations provided
**OTHER INFORMATION**			
Registration and protocol	24a	Provide registration information for the review, including register name and registration number, or state that the review was not registered.	Not reported - Missing: registration information
	24b	Indicate where the review protocol can be accessed, or state that a protocol was not prepared.	Not reported - Missing: registration information
	24c	Describe and explain any amendments to information provided at registration or in the protocol.	Not applicable - No registration or protocol mentioned
Support	25	Describe sources of financial or non-financial support for the review, and the role of the funders or sponsors in the review.	Funding section - "This research received no external funding"
Competing interests	26	Declare any competing interests of review authors.	Conflicts of Interest section - "The authors declare no conflicts of interest"
Availability of data, code and other materials	27	Report which of the following are publicly available and where they can be found: template data collection forms; data extracted from included studies; data used for all analyses; analytic code; any other materials used in the review.	Data Availability Statement - "Data supporting the reported results are available on request from the corresponding author. The data are not publicly available due to privacy and ethical restrictions."

PRISMA

PRISMA 2020 Checklist

From: Page MJ, McKenzie JE, Bossuyt PM, Boutron I, Hoffmann TC, Mulrow CD, et al. The PRISMA 2020 statement: an updated guideline for reporting systematic reviews. BMJ 2021;372:n71. doi: 10.1136/bmj.n71.
This work is licensed under CC BY 4.0. To view a copy of this license, visit https://creativecommons.org/licenses/by/4.0/
